# Assessment of earthquake-induced landslide inventories and susceptibility maps using slope unit-based logistic regression and geospatial statistics

**DOI:** 10.1038/s41598-021-00780-y

**Published:** 2021-10-29

**Authors:** Badal Pokharel, Massimiliano Alvioli, Samsung Lim

**Affiliations:** 1 School of Civil and Environmental Engineering, The University of New South Wales, Sydney, Australia; 2 Istituto di Ricerca per la Protezione Idrogeologica, Consiglio Nazionale delle Ricerche, Via Madonna Alta 126, 06128 Perugia, Italy

**Keywords:** Natural hazards, Geomorphology

## Abstract

Inventories of seismically induced landslides provide essential information about the extent and severity of ground effects after an earthquake. Rigorous assessment of the completeness of a landslide inventory and the quality of a landslide susceptibility map derived from the inventory is of paramount importance for disaster management applications. Methods and materials applied while preparing inventories influence their quality, but the criteria for generating an inventory are not standardized. This study considered five landslide inventories prepared by different authors after the 2015 Gorkha earthquake, to assess their differences, understand the implications of their use in producing landslide susceptibility maps in conjunction with standard landslide predisposing factors and logistic regression. We adopted three assessment criteria: (1) an error index to identify the mutual mismatches between the inventories; (2) statistical analysis, to study the inconsistency in predisposing factors and performance of susceptibility maps; and (3) geospatial analysis, to assess differences between the inventories and the corresponding susceptibility maps. Results show that substantial discrepancies exist among the mapped landslides. Although there is no distinct variation in the significance of landslide causative factors and the performance of susceptibility maps, a hot spot analysis and cluster/outlier analysis of the maps revealed notable differences in spatial patterns. The percentages of landslide-prone hot spots and clustered areas are directly proportional to the size of the landslide inventory. The proposed geospatial approaches provide a new perspective to the investigators for the quantitative analysis of earthquake-triggered landslide inventories and susceptibility maps.

## Introduction

Preparation of landslide inventories after a triggering event is a fundamental procedure to analyze and assess ground effects in the area hit by an earthquake^1,2^, providing information on the extent and magnitude of the landslide event^3^. Landslide hazard and risk assessment depend on landslide inventory maps (LIMs)^4^, as does the statistical study on the spatial distribution of landslides and susceptibility assessment^5^. The consistency of the inventory maps is dependent on their quality^6^. The completeness of inventory, the mapping unit used to classify landslide susceptibility, and the sampling balance between inventories are primary factors governing the reliability of landslide susceptibility maps (LSMs)^7^. Conclusive criteria to generate LIMs of earthquake-induced landslides have never been formalized^8^. However, studies exist about methods to reduce errors during photointerpretation procedures ^9^ and about standards to properly select images for the purpose^10^. Therefore, a careful analysis of completeness of inventories, and the quality of LSMs based on the inventories, can determine the degree of the usefulness of the inventories for various applications.

Standard criteria to define the quality and completeness of the inventories have not yet been established, partially due to the inadequacy or lack of metadata^6,11^. Nevertheless, some attempts have been made to assess the completeness of the inventories connected with the same earthquake event^12,13^. Existing studies suggest that the common methods of comparing inventories are based on visual analysis and statistical approaches. At the same time, there has been no rigorous analysis aimed at understanding the inventories of the same earthquake event but generating different LSMs. This paper focuses on performing a statistical and geospatial comparative analysis on the inventories and the LSMs obtained from the inventories using the standard classification methods.

Landslide susceptibility maps contain information on the relative spatial probability for landslides occurrence, depending on terrain conditions and the overall setting of area^14^. In general, LSMs may be prepared using qualitative and quantitative methods^15,16^. While qualitative methods determine the susceptibility level in a descriptive form based on the expert’s judgement, quantitative methods apply mathematical and statistical relationships between the landslide occurrence and predisposing factors for assessing the probability of landslide occurrence^17,18,18^ for earthquake-triggered landslides. The available literature suggests that, although many quantitative methods have been widely used to prepare LSMs^15^, there is no standard method to do so. A multi-variate quantitative method known as logistic regression (LR) is a data-driven and practical approach to analyze the presence or absence of landslides^18^. LR has been commonly used to assess the landslide occurrence probability and study event-based landslides^14,19^.

The selection of an appropriate digital elevation model (DEM) is an essential step in preparing quantitative LSMs^20^. The choice of independent factors, particularly the morphometric parameters derived from DEMs, influences the accuracy of LSMs^21^. The optimization of the factors can help enhance the accuracy of the susceptibility models^22^. Furthermore, the selection of the mapping unit for LSMs is vital because the accuracy of the data must match the partitioning of the mapping unit^23^. Grid cells are primarily used to evaluate and assess landslide susceptibility, but they neglect the physical boundaries of slopes. Instead, slope units (SUs) are closely related to the geological and topographic environment and are more suited for landslide zonation studies^24–26^. The devastating earthquake of magnitude 7.8 at Gorkha, Nepal, in 2015 and its aftershocks triggered nearly 25,000 landslides in the central Nepal Himalayas^27^. In this study, we analyzed five landslide inventories prepared manually after the Gorkha Earthquake 2015. First, we performed a quantitative comparison on these inventories by calculating an error index and analyzing landslides' distribution patterns with respect to the earthquake epicenter and major thrust systems, to reveal apparent differences. Second, we assessed the differences associated with morphometric factors by applying different sampling techniques to calculate their statistical significance, followed by calculating the performance of susceptibility maps generated within LR using these explanatory variables. Third, we applied geospatial analysis to investigate variations in the spatial clustering of the susceptibility with respect to the different inventories. The paper is organized as follows: Sect. 2 describes the available data, particularly the five landslide inventories analyzed here. Section 3 describes the methods adopted for the comparison, both of inventories themselves and of the corresponding LSMs. Results are presented and discussed in Sect. 4. Section 5 draws conclusions of this study.

## Data

The epicenter of the Gorkha Earthquake 2015 is located nearly 80 km northwest of Kathmandu Valley, 28.23° N latitude and 84.73° E longitude^28^. Earthquake aftershocks were scattered in the upper section of the anticlinorium system of the main central thrust (MCT)^29^. The strongest aftershock of magnitude 7.3 occurred on May 12, 2015, in the Dolakha district, approximately 140 km east of the mainshock epicenter^28^. Fourteen districts in the central Nepal Himalayas were the worst affected by the earthquake. Many scholars^30–33^ have conducted studies on the size, spatial distribution, landslide susceptibility and damage assessment with the aid of satellite images. A few of them prepared landslide inventories immediately after the earthquake, while others were compiled afterwards. The researchers applied different techniques for the preparation of their inventories.

**Figure 1:**
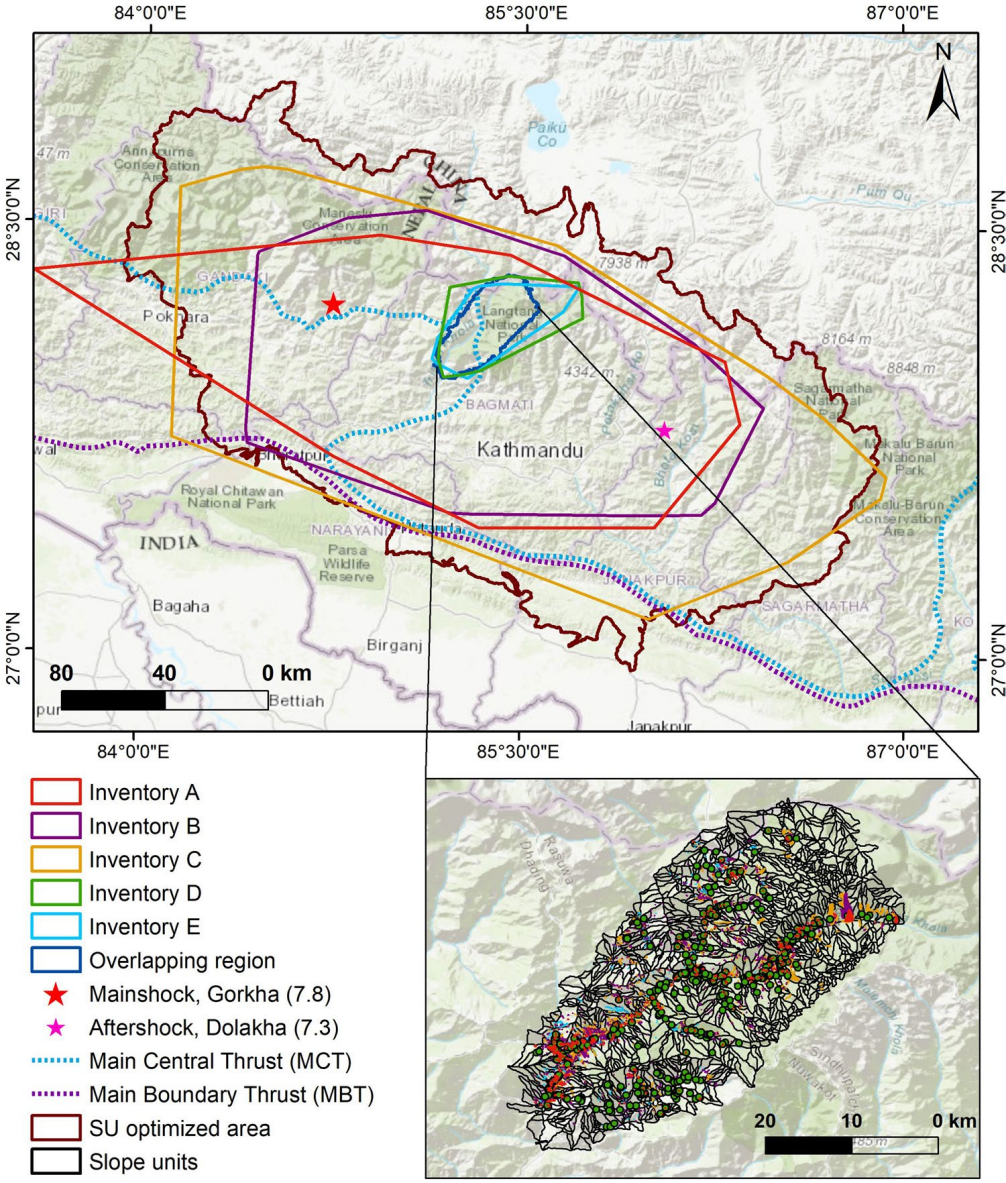
Map of the study area showing the extent of inventories considered in this work, overlapping region, earthquake epicenter and major thrust systems; thrust system modified after Stocklin^36^. The base layer is “World Topograhic Map” available as ArcGIS Online basemap (https://www.arcgis.com/). The map was created using ArcGIS version 10.8.1 (https://www.esri.com/).

We investigated five existing inventories over the impacted region produced by Zhang et al.^34^, Gnyawali et al.^32^, Roback et al.^33^, Kargel et al.^31^, Pokharel and Thapa^35^ at different times after the earthquake event. These five inventories are referred to as Inventories A, B, C, D and E, respectively. The overlapping region of the five inventories covers a section of Rasuwa, Nuwakot and Dhading districts (Fig. 1 and Table 1). It occupies an area of 1948 km^2^_,_ where elevation ranges from 356 to 7916 m. It comprises most of the Trishuli River watershed.

**Table 1:** Details of landslide inventories in an overlapping region considered in this work. Inventory D contains point locations of landslides; hence the landslide area is undefined.

Inventory	Number of landslides	Number of unstable SUs	Landslide area [km^2^]	Landslide area (%)
A	2075	198	4.11	0.47
B	1780	143	9.92	1.15
C	2118	132	14.57	1.69
D	371	204	–	–
E	359	74	10.92	1.26

The five inventories considered in this work are as follows.

### Inventory A

Zhang et al.^34^ mapped landslides triggered by the mainshock and aftershocks sequence. They used Gaofen-1 and -2 images and delineated 2645 landslides, represented by polygons in the inventory, employing pre-event and post-event analysis from the satellite images.

### Inventory B

Gnyawali and Adhikari^32^ produced a comprehensive polygon-based landslide inventory of 17,638 landslides in central Nepal (20,500 km^2^) using high-resolution optical satellite images available from Google Earth (GE). They considered landslides triggered by the main shock and aftershock sequence, occurred before the beginning of the next major monsoon.

### Inventory C

Roback et al.^33^ used very high-resolution satellite images, including DigitalGlobe WorldView-2 and -3, with a spatial resolution ranging from 30 to 50 cm. Most of the images were acquired between May 2 and May 8, 2015, in the Greater and Lesser Himalayas of China and Nepal. They mapped 24,915 polygons corresponding to landslides triggered by the earthquake between April 26 to June 15, 2015, in central Nepal.

### Inventory D

Kargel et al.^31^ implemented satellite-based techniques to investigate the landslides in the damaged region of central Nepal and Tibet. They used high- and medium-resolution satellite imagery (DigitalGlobe, NASA imageries, Landsat 8, WorldView, and others). Additional secondary data from media, photographs taken by locals, and helicopter-based assessments were also used. The inventory consists of 4312 point-like landslide locations.

### Inventory E

Pokharel and Thapa^35^ used 1.5 m pan-sharpened SPOT-5 satellite images acquired before Aril 2015 and freely available satellite images available after April 2015 to prepare a polygon-based landslide inventory in Rasuwa district (1544 km^2^), including 1416 polygons. Most of the landslides were delineated using the images acquired in May 2015, before the monsoon.

## Methodology

**Figure 2:**
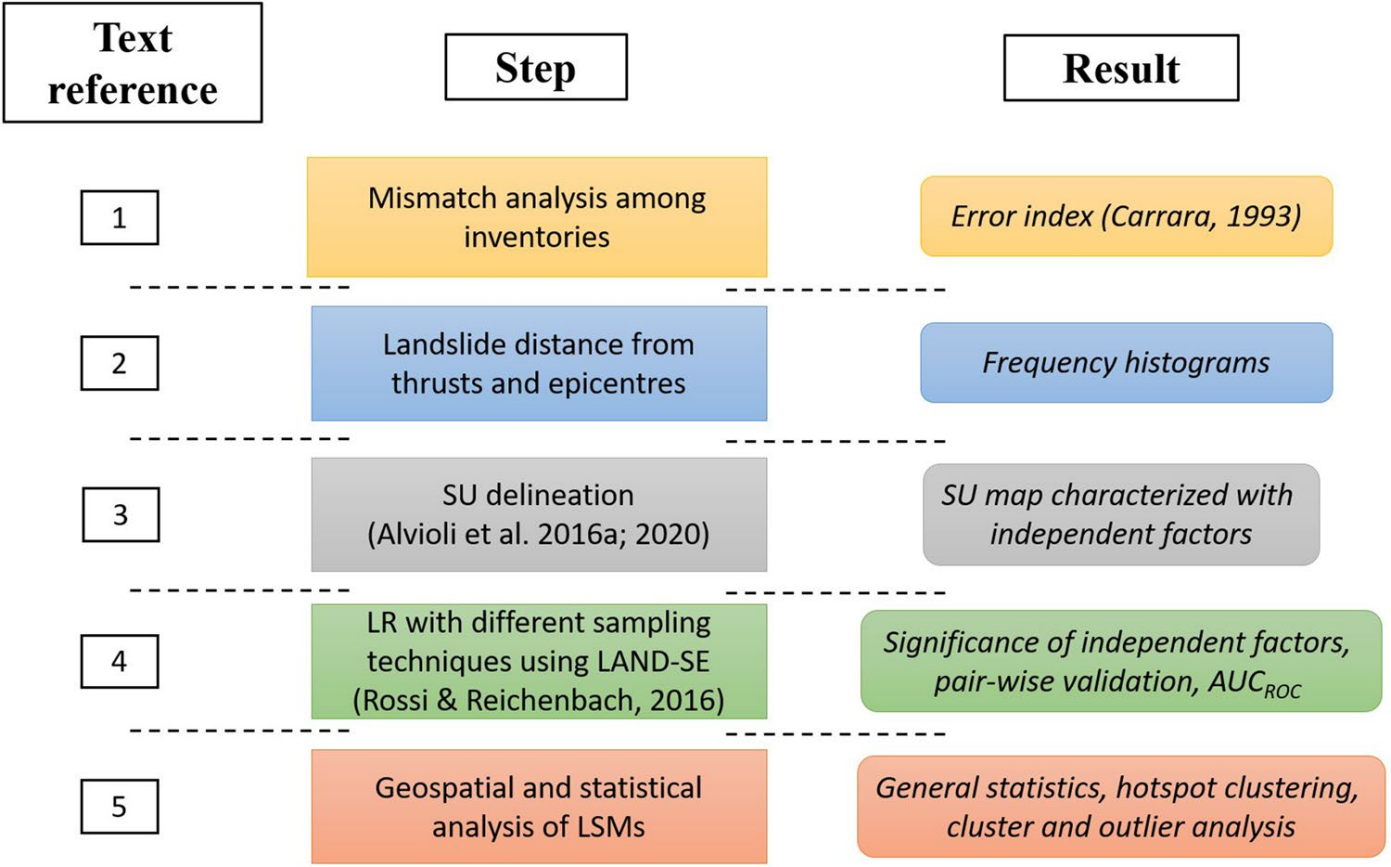
An illustration of the steps performed in this work to compare different inventories and the corresponding LSMs (*cf.* Section 3).

This work aims at performing a pairwise comparison among the landslide inventories to highlight their differences and their role in landslide susceptibility mapping within the LR method. The overall methodology is illustrated in Fig. 2 and consists of five steps: (1) calculation of a straightforward comparison index; (2) characterization of inventories with the distance of individual landslides from the epicenters and faults; (3) delineation of an SU map and characterization of each SU with morphometric and ground shaking variables; (4) calculation of SU-based LSMs for each inventory in the overlapping region and (5) calculation of SU-based LSMs in considerable substantially larger extent, for polygon-based larger inventories. Details of the five steps are as follows.

Step 1. An overlapping region among five inventories was considered to perform the comparison. The error index *E*_*I*_ proposed by Carrara^37^, and recently used by Alvioli et al.^38^ and Fiorucci et al.^10^, helped quantitatively comparing pairs of inventories in the overlapping region. The index is a quantitative estimate of the difference between two polygon-based inventories in a specific geographical region. It is defined as follows:

1 $${E}_{I}=\frac{{A}_{\cup }-{A}_{\cap }}{{A}_{\cup }} ,$$

where ${A}_{\cup }$ is the area occupied by either of the two inventories (individual landslide polygons), while ${A}_{\cap }$ is the area in common between the two inventories. Meena and Piralilou^12^ used a similar method. In Eq. (1), the symbols ∪ and ∩ represent the spatial GIS union and intersection, respectively; thus, they have spatial meaning and are meant to compare the pair of inventories under investigation pixel by pixel. The resulting error index *E*_*I*_, thus, is zero for two exactly overlapping inventories (i.e., if each polygon is exactly overlapping), and it is equal to unity for two completely non-overlapping inventories.

Step 2: We calculated the distance of individual landslides from the epicenters of the mainshock and of the biggest aftershock, from the Main Central Thrust (MCT) and the Main Boundary Thrust (MBT). We plotted the frequency (normalized histograms) of such values for all the inventories in the common area, and for the three larger inventories in the extended area.

Step 3: We adopted the r.slopeunits software developed by Alvioli et al.^39^ and the optimization algorithm of Alvioli et al.^40^ to generate an SU map that covers all of the inventories. The total number of SUs on the map is 91,947. The software r.slopeunits is a GRASS GIS module and is freely available (http://geomorphology.irpi.cnr.it/tools/slope-units). All the morphometric variables referred to in this work were calculated from the Cartosat-I DEM, at 30 m resolution. A freely available dataset published by the United States Geological Survey (USGS) was utilized to obtain the dynamic (ground-shaking) variables^41^.

Step 4. Landslide susceptibility assessment consists in classifying each mapping unit with a probabilistic index based on the knowledge of a dependent variable (here, landslide presence/absence) and a set of independent variables. Such classification can be conducted using many statistical and/or machine learning approaches such as LR, weight of evidence, frequency ratio, neural network, random forest, and others^42–44^. Logistic Regression is widely used to assess the spatial relation of landslide and their casual factors^45–47^. Hence, we selected LR to obtain LSMs corresponding to the five inventories considered in this study.

The relation between the occurrence of the phenomenon and independent variables is given by^45^:

2 $$p=\frac{1}{1+{e}^{-z}} ,$$

where $p$ is the chance of phenomena (here, probability of landslide occurrence) and $z$ is a linear combination of independent variables (here, predisposing factors). The linear combination in Eq. (2) reads as follows ^45^:

3 $$z={b}_{0}+{b}_{1}{x}_{1}+{b}_{2}{x}_{2}+\dots +{b}_{n}{x}_{n} ,$$

where ${b}_{0}$ is the intercept of the linear model, ${b}_{i}$ (*i* = 0, 1, 2, …, n) represent the slope coefficients of the regression model and ${x}_{i}$ (*i* = 0, 1, 2, …, n) represent the independent variables (*i.e.*, landslide predisposing factors).

Each SU was characterized by the presence or absence of landslides from each of the five inventories and descriptive statistics (mean and standard deviation (S.D)) of independent morphometric variables^7,48,49^ (Table 2). In addition, we considered landforms classes obtained with the r.geomorphon software in GRASS GIS^50^. Ridge, spur, slope, and hollow were selected as the landform classes, and we characterized each SU with the percentage of each class. The morphometric variables used here have direct interpretation regarding their impacts on landslide occurrence^7^. Following Tanyas et al.^7^, we did not include thematic variables such as land use and/or geology in the LR classification. We cannot clearly explain their effect because we did not distinguish between different kinds of landslides.

**Table 2:** List of independent variables as studied in Tanyas et al.^7^ and adopted here. We mentioned the GRASS GIS modules used to calculate each variable at grid cell level, from which mean and S.D were subsequently obtained at SU level.

Type	Independent variables	GRASS GIS module, or reference
Dynamic	PGA	USGS^41^
Dynamic	PGV	USGS^41^
Dynamic	MMI	USGS^41^
Static	Slope	r.slope.aspect
Static	Topographic Wetness Index (TWI)	r.topidx^51^
Static	Vector ruggedness measure (VRM)	r.vector.ruggedness^52^
Static	Local relief	r.neighbors^50^
Static	Landform classes	r.geomorphon^50^
Static	Plan curvature (PlanC)	r.slope.aspect
Static	Profile curvature (ProfC)	r.slope.aspect

Dynamic variables of the problem are ground-shaking parameters, for which we calculated average values within each SU as well. Specifically, we used peak ground acceleration (PGA), peak ground velocity (PGV) and modified Mercalli intensity (MMI). These variables, at variance with morphometric variables used in this work, are specific to the earthquake event^7^.

**Figure 3:**
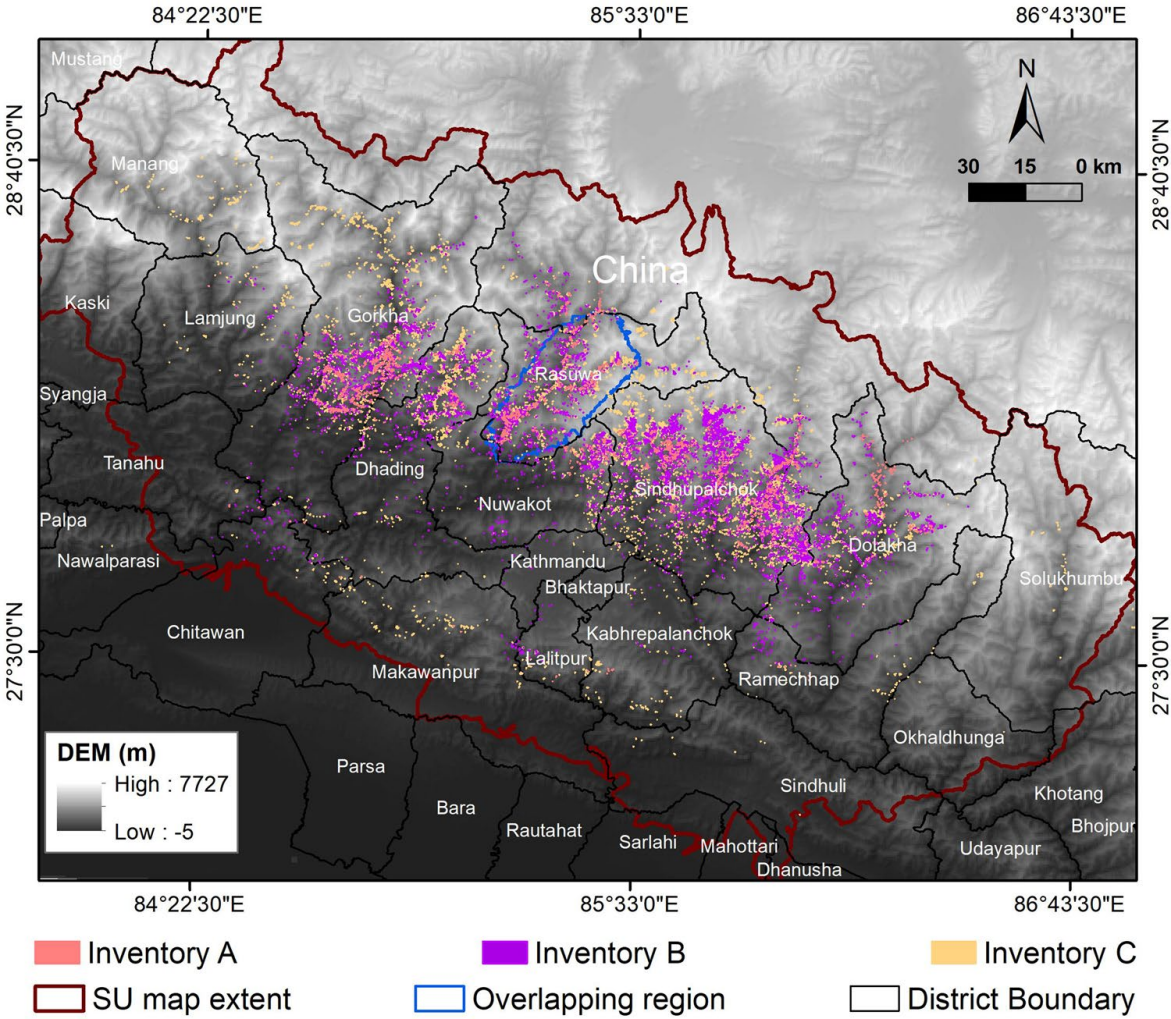
Extent of the three larger inventories (**A**–**C**). The underlying elevation is the Cartosat DEM (https://bhuvan.nrsc.gov.in). The map was created using ArcGIS version 10.8.1 (https://www.esri.com/).

Classification of each SU using LR, within the overlapping region for all the five inventories, and within the larger extent shown in Fig. 3 for the three larger inventories, required a training step and a validation step. Training and validation were performed using two independent (different) samples, *i.e.,* two subsets of the SU map.

To train the LR model we obtained, for the overlapping region, the smallest number among the stable and unstable SUs among all the inventories and generated the training samples as follows. This corresponds to 74 unstable SUs, dictated by Inventory E. A random selection of 75% of such unstable SUs and an equal number of stable SUs for each landslide inventory represented one instance of the training sample. The random selection was repeated 20 times for each inventory to obtain a range of results. LR was applied to the 20 training samples using the glm() function (an implementation of the generalized linear model) within the R language. Then, to calculate the p-values for the independent variables, we run a χ-square test in the 20 training runs. Moreover, we run a pairwise collinearity test among the variables and ones with a value larger than 0.7 in the correlation matrix were discarded (S.D of VRM and mean of profile curvature).

The significant landslide predisposing factors were analyzed using p-values for each inventory in the common region. We calculated the area under the curve of the receiving operating characteristic (AUC_ROC_) for each training sample and performed pairwise validation between the inventories on an independent sample. The validation sample consisted of 20 runs with randomly selected slope units of the second inventory of each pair, as for the training step. For each training and validation run, we calculated the mean and standard deviation of AUC_ROC_.

In addition, the success rate of the LSMs in the common area was examined by calculating AUC_ROC_ for each inventory by training the LR model them with three different strategies. The first strategy (TR1): for each inventory, we selected 70% of stable (or unstable, whichever was smaller among all inventories) slope units and an equal number of unstable (or stable) as a training sample, and the remaining 30% as a validation sample. The second strategy (TR2): we selected the smallest number between stable and unstable slope units to train the LR model. Specifically, the total number of stable and unstable slope units for Inventory E are 319 and 1338, respectively. Hence, the training sample contained 319 stable and 319 unstable SUs, for all the inventories. Third strategy (TR3): all the SUs in the map were used as the training sample; no validation is implied. The sampling with strategies TR1 and TR2 were repeated 20 times to obtain a range of results.

Step 5: Inventories A, B and C were used to prepare LSMs in the larger area shown in Fig. 3, while the two smaller inventories, D and E, were neglected in this step. We adopted the LAND-slide Susceptibility Evaluation (LAND-SE) software by Rossi and Reichenbach^49^, making landslide susceptibility zonation easy; we selected the LR method for this step of the four possible classification methods contained in the software. Thus, we obtained three LSMs, *i.e.*, a probabilistic landslide susceptibility value for each SU.

Eventually, we applied three geo-statistical tests on the LSMs obtained from the larger inventories. The first test evaluated the basic statistics: mean and standard deviation. The second test compared the Pearson and Spearman’s correlation. The third test aimed at a geospatial analysis with Cluster and Outlier Analysis (Anselin Local Moran’s I) and Hot Spot Analysis (Getis-Ord Gi*). Getis-Ord Gi*, a family statistic introduced by Getis and Ord^53^, have been used by scholars to determine spatial patterns. For example, to detect extremely slow-moving landslides, Lu et al.^54^ introduced Persistent Scatterers Interferometry Hotspot and Cluster Analysis (PSI-HCA) and applied Getis-Ord Gi* statistics to run this approach. They evaluated the clustering level of Persistent Scatterers. The literature^55–57^ shows that GIS-based applications like hotspot analysis (Getis-Ord Gi*), and cluster and outlier analysis based on Anselin local Moran’s I, can be used as a tool to produce groups/clusters using spatial autocorrelation at the local level. In this study, the goal was analyzing the clustering pattern of susceptibility, represented by a single value in each polygon and each map. We considered the three LSMs pairwise for these geospatial tests and subtracted their values in each SU as *A-B*, *B-C* and *C-A,* respectively^58^. This implies the comparison values range from − 1 to 1.

## Results and discussions

### Error index

**Figure 4:**
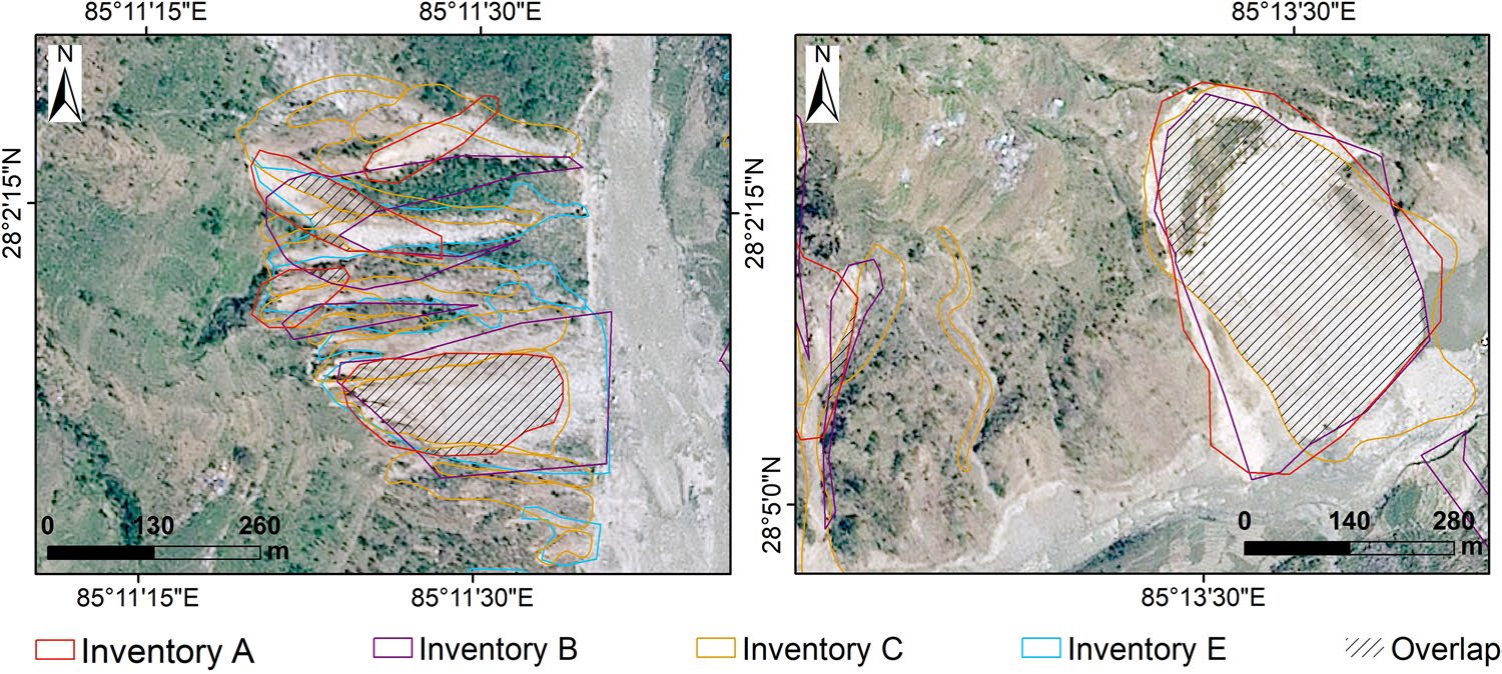
Landslides mapped (examples in two figures) by different authors after the Gorkha Earthquake. The satellite image is SPOT-7, taken on May 03, 2015, provided by AIRBUS DS. Inventory (**A**–**C**) are available on https://www.sciencebase.gov/catalog, Inventory D is available on https://rds.icimod.org/. and Inventory E was prepared by the first author. This figure was created using ArcGIS version 10.8.1 (https://www.esri.com/).

Table 3 lists values of the error index, Eq. (1), for the overlapping region of polygon-based inventories: error indices are greater than 0.5 for all pair of inventories, denoting relatively poor overlap between all of the inventories. Figure 4 shows two details of the area, to illustrate the different mapping styles of different authors.

**Table 3:** Error index defined in Eq. (1), after Carrara et al.^37^, Alvioli et al.^38^ and Fiorucci et al.^10^, calculated for the four polygon-based inventories in the overlap region. A smaller error index denotes better spatial agreement between the corresponding pair of inventories.

	Inventory A	Inventory B	Inventory C
Inventory B	0.8	–	0.73
Inventory C	0.87	0.73	–
Inventory E	0.9	0.85	0.86

In general, mismatches among the inventories may occur for several reasons, *e.g.*, the difference in scales of base maps, the study's objective, type of photographs or satellite images used, the extent of a field study, skills of the interpreter, etc^9–11,59^. Xu et al.^60^ emphasized the quality of landslide inventories influenced the volumetric analysis of earthquake-triggered landslides resulting in substantial errors in their calculation. Valagussa et al.^61^ reported uncertainty in the landslide volume calculation of the Gorkha Earthquake triggered landslides related to the quality and completeness of the inventories. The images used by Inventory C were of very high resolution (30–50 cm), and they were acquired right after the event and before the monsoon, which implies that the images were free of cloud coverage. The areal extent surveyed by the authors is greater than the other four inventories. The larger number of landslides is not necessarily a conclusive criterion to assess completeness of an inventory. In manual or semi-automatic delineation, the interpreter might misjudge the barren/non-landsliding region as landsliding. The mismatch in the sample locations in Fig. 4, and the results of the error index in Table 3, suggest that the authors might have considered landslide bodies in different ways, for one or more of the reasons hypothesized above.

### Distance from epicenter and thrusts

**Figure 5:**
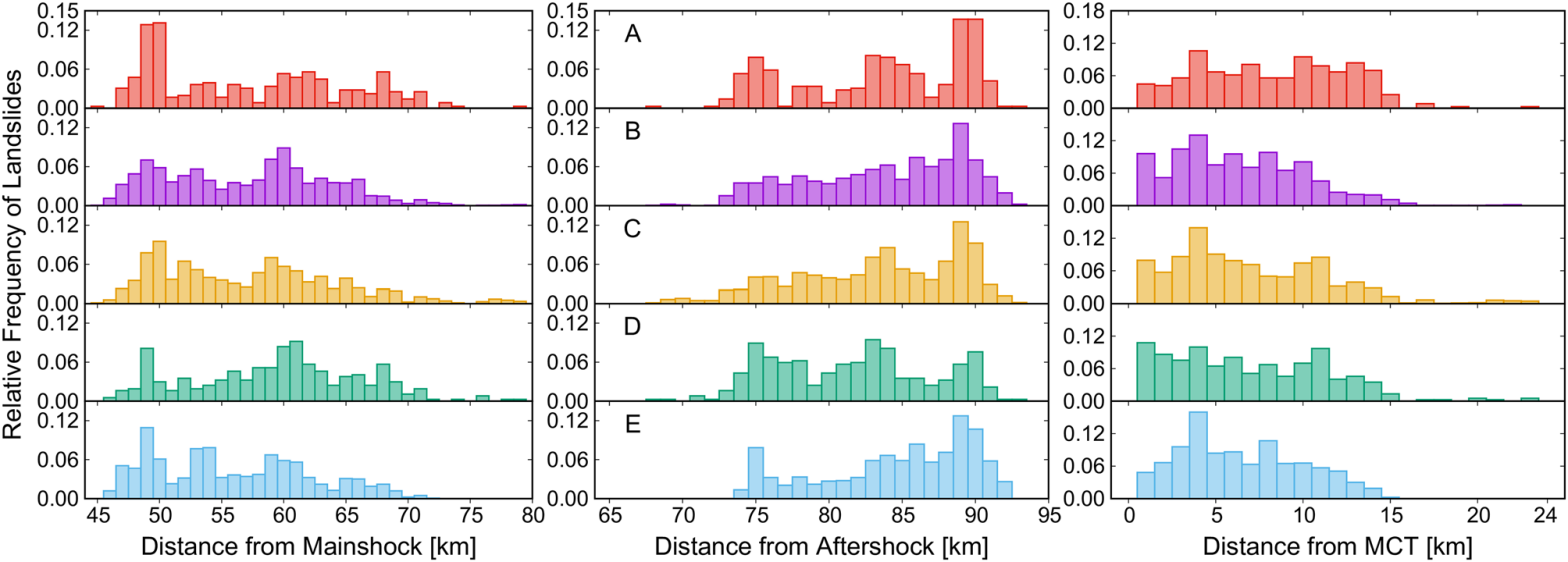
Relative frequency of landslides distance from the mainshock epicenter (left column), from the aftershock epicenter (center column) and the main central thrust (right column), for the overlapping region of all the inventories (**A**–**E**) considered in this work (*cf.* Figure 1).

We have presented normalized histograms (frequencies) of the distance of individual landslides from the mainshock epicenter, most significant aftershock epicenter, and MCT within the overlapping area of the five inventories considered in this work (Fig. 5). Inventory A has the highest peak of landslide frequency (> 0.12) for the mainshock, in the distance range 45–50 km. The peak for Inventory C and Inventory E falls in the same distance range. On the other hand, Inventory B and Inventory D show a peak at 60 km from the mainshock epicenter. For the aftershock, the maximum frequency of landslides for Inventory D is in the distance range 70–75 km, whereas for all of the remaining inventories the peak lies in the distance range of 85–90 km.

All the inventories, except Inventory D, have the highest number of landslides clustered in the distance range 3–5 km from MCT, whereas Inventory D does not show a clear peak. The frequency for Inventory D is high in about 0–2 km and 10–12 km from MCT. The overall trend shows that the number of landslides decreased gradually with the increase in distance. Inventory A does not show a clear peak in the frequency plot of distances from MCT, in Fig. 5.

**Figure 6:**
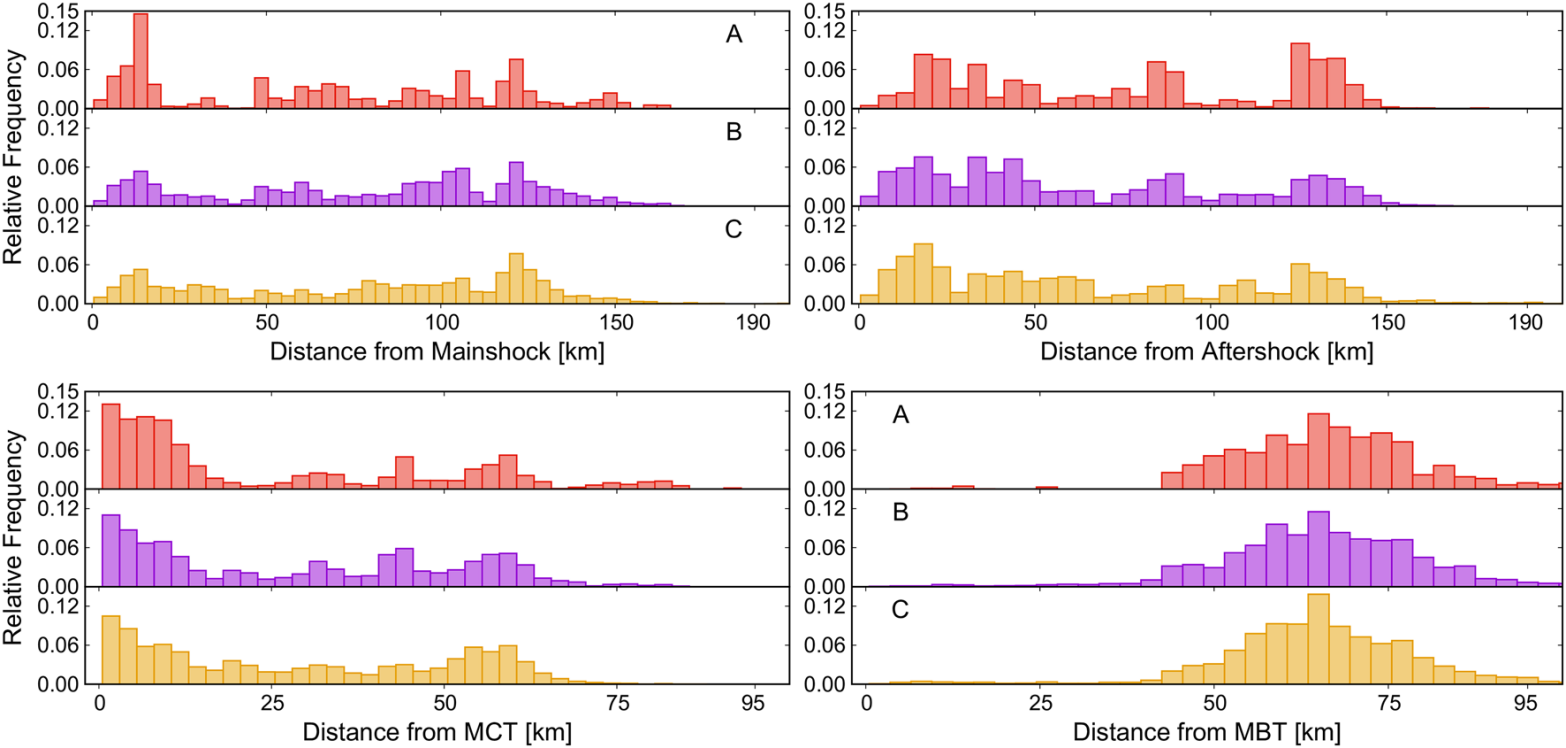
Relative frequency of landslides distance from the mainshock epicenter (top row, left column), from the aftershock epicenter (top row, right column), the main central thrust (bottom row, left column) and from the main boundary thrust (bottom row, right column). Plots correspond to the larger extent of the three bigger inventories considered in this work (*cf.* Figure 3).

We looked at similar plots for three larger inventories (Fig. 6). Both MCT and the MBT as a reference thrust system. MBT was not included in the overlapping region because it was far off. Inventory B and Inventory C show the maximum frequencies in the distance range 100–150 km from the mainshock epicenter. For Inventory A, there is a very high frequency in the distance range of 20–30 km. The peak for this inventory is distinctly high in comparison with the other two. The difference in the peak frequency (at the distance range of 100–150 km) for Inventory A is similar for the aftershock epicenter. Inventory B does not show a specific peak, whereas, for Inventory C, the maximum frequency lies near the epicenter of aftershock. For both MBT and MCT, the overall trend of graphs for all the three inventories is similar. Landslides are clustered closer to MCT as compared to MBT.

The frequency plots of distance from epicenter and major fault systems, limited to the overlapping region, do not show substantial differences except for the case of Inventory A. The research team who mapped Inventory A focused on the region close to and around the event's epicenter, for a very distinct rise exists in the number of landslides in this area. This supports the statement that inventories may differ from each other depending on the objective of mapping. The dense cluster of landslides near the main central thrust (MCT) implies that the thrust system near the epicenter (here, MCT) is highly prone to landslides. This is supported by the studies conducted by Nepal et al.^62^ and Shrestha et al.^63.^

### Significance of causal factors and success rate

**Figure 7:**
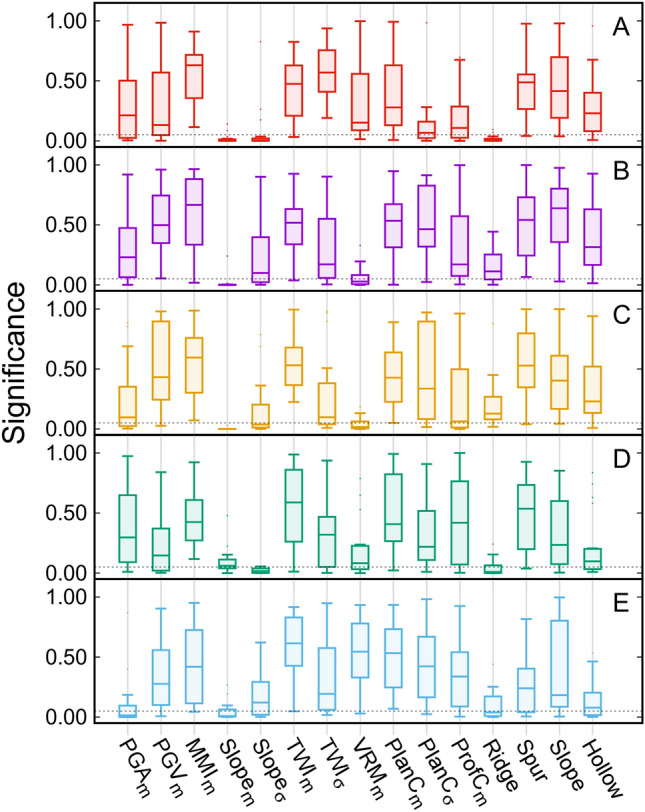
Significance of the different variables in terms of the p-value in LR, obtained from 20 iterations of the general linear model for each landslide inventory, initialized with corresponding random selections of the training sample. The horizontal dashed line represents the significance threshold adopted here (0.05).

As explained in Step 4 (*cf.* Section 3), boxplots in Fig. 7 help show the results of p-values associated with each inventory, stemming from the 20 runs of LR trained with different random selections of SUs. VRM (S.D) and profile curvature (S.D) were discarded following the collinearity test. The variables with p-value less than 0.5 were considered as significant factors. Slope (mean) followed by ridge landform were substantial for most of the inventories. This implies that slope is very relevant for earthquake-induced landslides^64^, as expected. The summarization of p-values of variables for each inventory is given in Supplementary Table S1.

**Figure 8:**
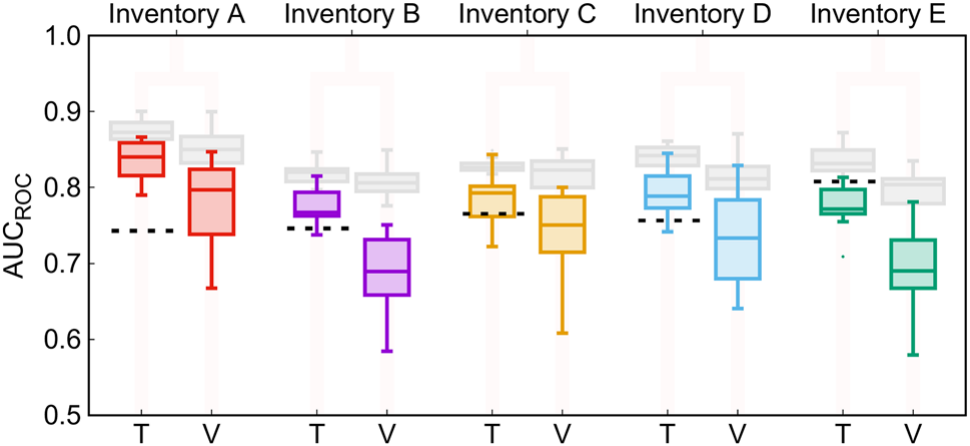
Boxplots showing AUC_ROC_ values for the five inventories in the overlap region considered in this work (*cf.* Figure 1). Boxplots in color correspond to training/validation strategy TR1; grey to TR2; dotted lines to TR3; see Sect. 3 for a description of the different strategies.

As mentioned in Step 4 (*cf*. Section 3 and Fig. 2), AUC_ROC_ values for all the inventories were plotted with three different sampling strategies (Fig. 8). The colored boxes in the figure represent the TR1 strategy (70% of the smallest number of stable or unstable SUs in each inventory as training sample and the remaining 30% in validation sample, 20 random selections). Grey boxes in Fig. 8 represents TR2 (smallest number of stable/unstable SUs across all of the five inventories, and the equal number of unstable/stable for training, the remaining in validation; 20 random selections) case and the dotted line represents TR3 case (all of the SUs to train the LR; thus, no validation and no variability of the results). For TR1, the Inventory A (mean of distributions ~ 0.85 for training and _~_0.80 for validation) outperforms the rest of the inventories. The validation performance value of Inventory B and E is smaller than 0.70, while for the others it ranges in 0.73–0.79. There is not much variance in the results for TR2 (0.80–0.85). For TR3, the performance is highest for Inventory E (approximately AUC_ROC_ = 0.80), and other inventories share similar values (0.73–0.75).

Pairwise validation of the LSMs from different inventories does not show distinct differences (Table 4). In most cases, for an individual inventory, the value of AUC_ROC_ is excellent when validation is performed using a sample extracted from the other inventories. Unlike our previous work^64^, the performance of the LSM obtained by applying LR does not show a dependence on the size of landslide inventory. We ascribe the difference in results to the use of relatively large SU polygons in Pokharel et al.^64^ and an optimized SU map containing much smaller polygons. Large SUs may have a reduced discrimination power to distinguish the presence/absence of many landslides highly clustered in space. This also implies that the quality of the base mapping unit (here, slope unit) affects the reliability of susceptibility maps.

**Table 4:** Pairwise validation among five inventories in the overlapping area. The table presents the mean with one standard deviation confidence level for the AUC_ROC_ obtained from each testing/validating pair, with 20-fold random selection.

	Validated by inventory					
		A	B	C	D	E
Trained with Inventory	A	**0.78 ± 0.03**	0.80 ± 0.03	0.82 ± 0.03	0.82 ± 0.04	0.80 ± 0.02
	B	0.83 ± 0.03	**0.74 ± 0.01**	0.80 ± 0.03	0.71 ± 0.02	0.80 ± 0.03
	C	0.83 ± 0.02	0.77 ± 0.02	**0.74 ± 0.03**	0.77 ± 0.02	0.79 ± 0.02
	D	0.88 ± 0.03	0.81 ± 0.03	0.81 ± 0.03	**0.71 ± 0.03**	0.80 ± 0.03
	E	0.83 ± 0.03	0.78 ± 0.03	0.80 ± 0.03	0.78 ± 0.03	**0.77 ± 0.02**

The values of AUC_ROC_ calculated from the different LSMs adopting different sampling techniques were also shown to have different average values and variability. All these points further exhibit that preparation of a landslide inventory is a subjective process. Henceforth, the derived LSMs are subjective as well and depend on many factors. Another possible source of differences in LSMs is the statistical or machine learning method used to calculate susceptibility values and classify the mapping units into susceptibility classes, which was not studied here since we only adopted LR. Moreover, Bordoni et al.^59^ emphasized that, for event-based landslide susceptibility (*e.g.,* rainfall), the susceptibility distribution depends on the landslide type and mapping techniques. In the inventories considered for this study, there is no differentiation in the types of landslides (shallow or deep-seated) and landslide zones (depletion and deposition area).

In addition, we stress that, considering five independently produced landslide inventories, we effectively performed a real validation—pairwise validation of LSMs produced by any of the inventories, validated four other independent inventories. This is seldom considered in the literature, because it is fairly rare to have data to support actual validation data: it is much more common to split all of the available data, typically collected at one time by the same method and investigator, into training and validation datasets—as we did, for example, to prepare Fig. 8.

### Comparison of landslide susceptibility maps (LSMs)

**Figure 9:**
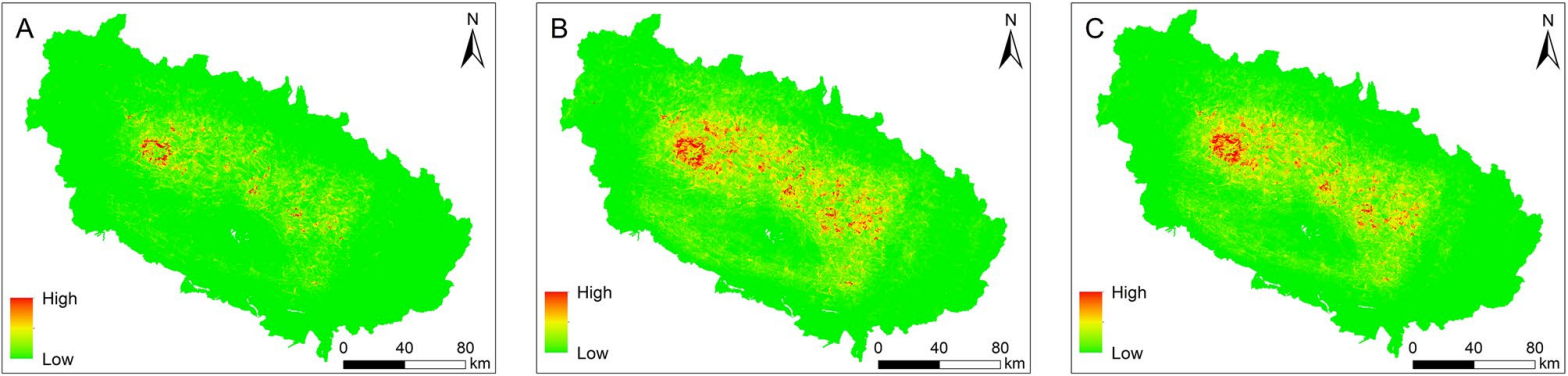
Landslide susceptibility maps obtained within the LR model for the three larger inventories (**A**–**C**) considered in this work (*cf.* Figure 3). 0 and 1 represent lowest and highest value of LS respectively. The maps were created using ArcGIS version 10.8.1 (https://www.esri.com/).

We prepared LSMs for three larger inventories (A, B and C), shown in Fig. 9. The range of possible landslide susceptibility (LS) values is [0,1], consistently with the interpretation of LS as a (relative) probability. Visual analysis shows that map B has the largest LS range. The high LS area has a more significant extent in map B, whereas the low LS area has a larger extent in map C. Table 5 lists the general statistical comparison; mean and S.D. Table 6 lists the values of the correlation between each pair of LSMs is performed using Spearman’s and Pearson’s correlation.

**Table 5:** General statistics of landslide susceptibility values, described in Sect. 3, for the LSMs from the three larger inventories considered in this work (*cf.* Figure 3); S.D stands for standard deviation.

Inventories	Mean	S.D
A	0.013	0.037
B	0.045	0.094
C	0.057	0.105

**Table 6:** Spearman’s and Pearson’s correlation coefficients between the three pair of LSMs from larger inventories A, B and C.

	Inventories		
	A *vs*. B	A *vs*. C	B *vs*. C
Spearman's correlation	0.9936	0.9976	0.9952
Pearson's correlation	0.9322	0.9217	0.9907

**Figure 10:**
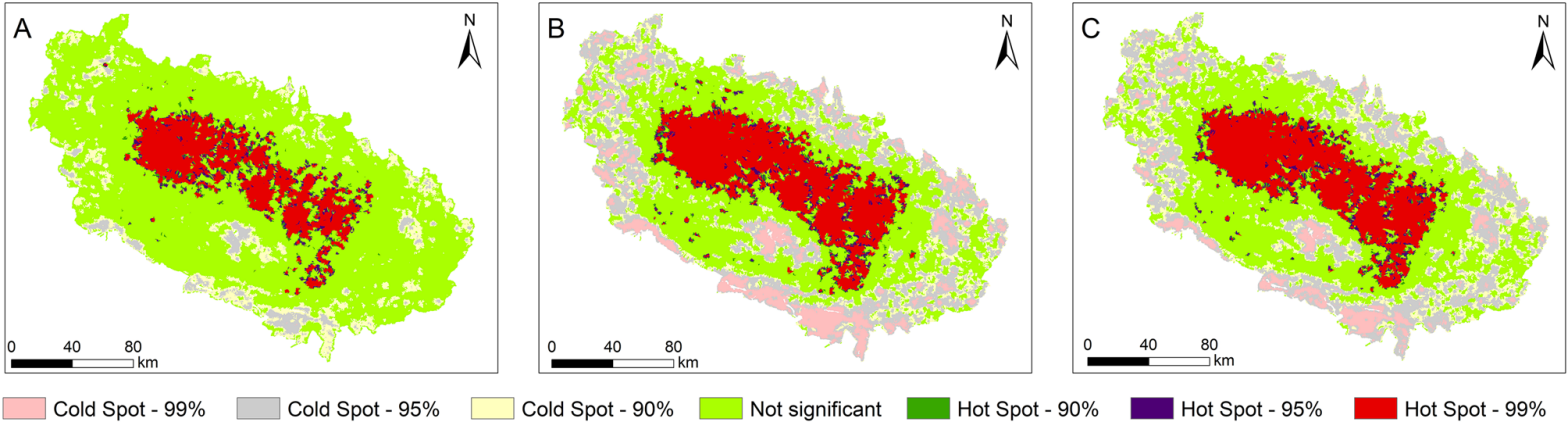
Hotspot analysis of LSMs for three inventories (**A**–**C**). Percentages represent confidence level, as produced by the hotspot analysis. The maps were created using ArcGIS version 10.8.1 (https://www.esri.com/).

As mentioned in Step 5 (*cf.* Section 3), for each pair of maps, one map was subtracted from the other to perform geospatial analysis. The results of the hotspot analysis of individual maps are presented in Fig. 10 and Table 7. The relevant results of this analysis for subtracted maps are shown in Supplementary Table S2.

**Table 7:** Results of hot spot analysis, described in Step 5 (*cf.* Section 3). For each map, corresponding to an individual inventory, we list the percentage of slope units in each hot spot class. The percentages in the “Result” column represent the confidence level.

Results	Inventories		
	A	B	C
Hot Spot 99%	0.004	15.1	1.1
Hot Spot 95%	6.4	23.5	2.1
Hot Spot 90%	16.3	10.3	9.0
Not Significant	62.9	31.6	13.2
Cold Spot 90%	1.2	1.2	15.3
Cold Spot 95%	2.0	2.2	26.1
Cold Spot 99%	11.14	16.9	33.2

None of the maps shares common characteristics of the hot spot and cold spot clusters. Map B has the largest hot spot cluster among the individual maps, and map A has the largest cold spot cluster. More than 60% of the area in map C belongs to non-significant clusters, which varies noticeably in the other maps. The variance is explicit in the clusters among the subtracted maps as well.

**Figure 11:**
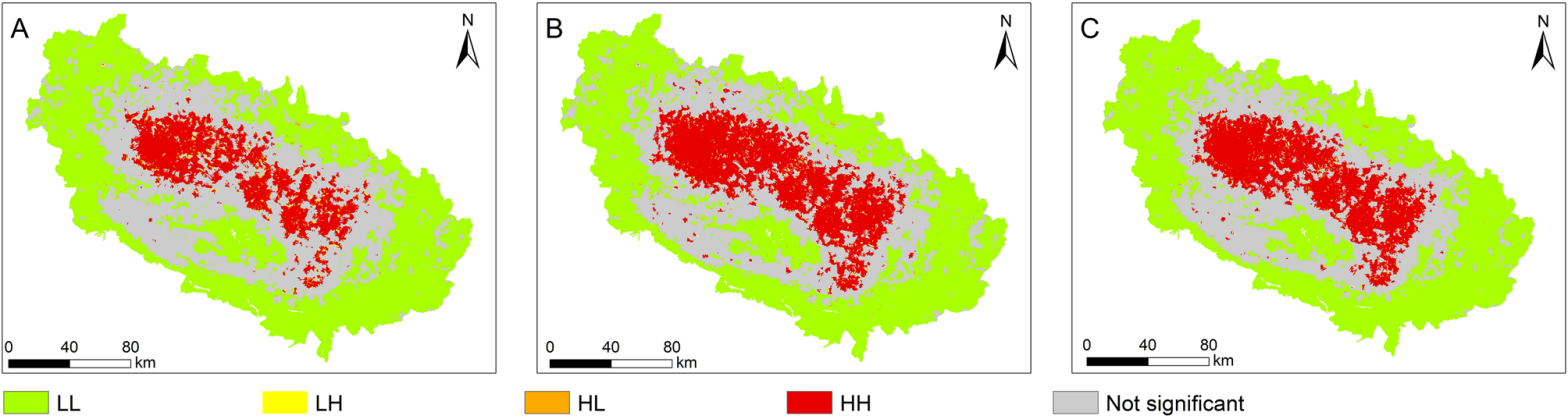
Cluster and outlier analysis of LSMs for three inventories (**A**–**C**). LL: a cluster of low values, HL: high outlier, LH: low outlier, HH: a cluster of high values. The map were created using ArcGIS version 10.8.1 (https://www.esri.com/).

Cluster and outlier analysis was performed with the same criteria as a complement to hot spot analysis, presented in Fig. 11 and Table 8. The high and low clusters result overlap with that of the hot spot. The non-significant area for this analysis is largest for map C, like in the hotspot analysis. As for the subtracted maps of hotspot analysis, a clear difference is observed between high cluster and low cluster in cluster and outlier analysis (Supplementary Table S3).

**Table 8:** Results of cluster and outlier analysis, for individual maps, described in Step 5 (*cf.* Section 3). The number represents the percentage.

Results	Inventories		
	A	B	C
Not significant	31.8	28.2	27.1
Cluster: High	10.5	15.6	14.8
High Outlier	0.1	0.3	0.1
Low Outlier	1.6	1.7	1.6
Cluster: Low	56.0	54.2	56.4

The general statistical comparison of LSMs from the three larger inventories shows that map from Inventory C, the largest, has the highest average value (0.05) of LS and standard deviation. However, the correlation coefficients are greater than 0.9 for all three cases (Table 6). On the other hand, the clustering analysis results show that map from Inventory C occupies the largest area among all inventories for “high cluster”. Map from Inventory A occupies the smallest area for the hot spot, cluster/outlier analysis. The non-significant area is large for map from Inventory A, which might be due to fewer mapped landslides. Overall, the geospatial analysis exhibits that the information to assess LSMs can be gathered by comparing LS values among maps obtained from different inventories. Further, the comparison can be facilitated by generating the hotspot clusters. The only use of statistical tests overestimates the correlation between the inventories and the performance of LSMs.

## Conclusions

Landslide inventories are the primary data source to prepare susceptibility, risk, and hazard zonation. Quality and completeness of an inventory are crucial parameters to be considered while implementing any zonation, but no standard technique exists to assess them. In the case that different inventories exist for a given area and/or a given triggering event, one can obtain LSMs generated from the inventories using the same independent variables and the same classification method. Comparison of LSMs is useful to understand how differences in the inventories themselves propagate to derivative maps.

In this study, five inventories prepared independently after the Gorkha earthquake by different geomorphologists were compared. The availability of these inventories allowed to conduct a thorough comparison among their contents and LSMs obtained thereof, using LR. Results of the study helped in outlining a) the statistical mismatches among the inventories and the reasons behind that, b) subjectivity in preparing landslide inventories and derived LSMs, and c) the importance of geospatial analysis in establishing the differences in the LSMs derived from different inventories prepared for the same event. This work can help researchers in prioritizing the objective of mapping and preparing LSMs based on earthquake events.

Results obtained in this study support the following conclusions:

i. Landslide inventories are descriptive products prepared by collecting data from aerial photographs, satellite images, field surveys. Hence, they are influenced by the quality of those data and the purpose of the effort. LSMs based on general-purpose inventories are not effective. Objective-based inventory preparation, possibly distinguishing landslide type, has the potential of improving the quality of LSMs.
ii. Selection of landslide predisposing factors and mapping unit to prepare LSMs are two critical steps. The performance of the LSMs also depends on different sampling techniques of training/validation samples and the availability of optimized mapping units—here, slope units.
iii. The results of hot spot, cluster and outlier analysis show that “high cluster” is dominant in the map from inventory with the highest number of landslides (Inventory C), whereas “non-significant” is dominant in the map from smallest inventory (Inventory A). The implication is that insufficient data might contribute to false or less reliable outputs. Hence, compilation of landslide inventories requires complete mapping, in the surveyed area.

In this work, the availability of several, independent landslide inventories for the same geographical area and the same triggering event allowed us direct comparison of the inventories themselves, and of susceptibility maps obtained from them. Though the availability of such multiple datasets is not a very common situation, it allowed us to show that the propagation of differences from the inventories to derived maps is not trivial, and it would be very difficult to spot in absence of an independent benchmark, and by simply calculating AUC_ROC_ values. The implication is that removing the subjectivity and attention to completeness in compilation of inventories is of paramount importance. Geospatial analysis provided a broader perspective, in combination with statistical tests, for the quality assessment of earthquake-triggered landslide inventories.

## Supplementary Information

Supplementary Information.
