# Supplementary material for: Assessment of earthquake-induced landslide inventories and susceptibility maps using slope unit-based logistic regression and geospatial statistics
Source: Sci Rep. 2021 Oct 29;11:21333. doi: 10.1038/s41598-021-00780-y (PMC8556321; doi:10.1038/s41598-021-00780-y)
Supplement: Supplementary file 1 — Supplementary Information. [file 41598_2021_780_MOESM1_ESM.docx]

**Assessment of earthquake-induced landslide inventories and susceptibility maps using slope unit-based logistic regression and geospatial statistics**

Badal Pokharel^1 *^, Massimiliano Alvioli^2^, Samsung Lim^1^

^1^School of Civil and Environmental Engineering, The University of New South Wales, Sydney, Australia

^2^Istituto di Ricerca per la Protezione Idrogeologica, Consiglio Nazionale delle Ricerche, via Madonna Alta 126, I-06128, Perugia, Italy

^*^ [b.pokharel@unsw.edu.au](mailto:b.pokharel@unsw.edu.au)

**Supplementary Table S1.** Summarization of the independent factors in the order of their increasing p-values for landslide inventories obtained from 20 random selections of the training/validation datasets used to initialize LR.

| **Inventories** | **Independent factors in order of increasing p-value** |
| --- | --- |
| A | Ridge (0.015), Slope_m_ (0.016), Slope_σ_ (0.069), PlanC_σ_ (0.13), ProfC_m_ (0.20), Hollow (0.28), PGA_m_ (0.29), VRM_m_ (0.30), PGV_m_ (0.31), PlanC_m_ (0.40), Spur (0.43), TWI_m_ (0.44), Slope (landform class) (0.46), MMI_m_ (0.55)_,_ TWI_σ_ (0.58) |
| B | Slope_m,_(0.013) VRM_m_ (0.060), Ridge (0.15), Slope_σ_ (0.24), PGA_m_ (0.28), TWI_σ_ (0.30), ProfC_m_ (0.31), Hollow (0.39), TWI_m_ (0.504), PlanC_m_ (0.509), PlanC_σ_ (0.515), Spur (0.516), PGV_m_ (0.52), MMI_m_ (0.56), Slope (landform class) (0.58) |
| C | Slope_m_ (0.0018)_,_ VRM_m_ (0.404), Slope_σ_ (0.149), Ridge (0.20), PGA_m_ (0.23), TWI_σ_ (0.265), ProfC_m_ (0.267), Hollow (0.35), PlanC_m_ (0.43), Slope (landform class) (0.44), PlanC_σ_ (0.45), PGV_m_ (0.49), MMI_m_ (0.5360), TWI_m_ (0.5367), Spur (0.539) |
| D | Slope_σ_ (0.021)_,_ Ridge (0.042), Slope_m_ (0.09), VRM_m_ (0.17), Hollow (0.20), PGV_m_ (0.24), PlanC_σ_ (0.323)_,_ Slope (landform class) (0.324), TWI_σ_ (0.326)_,_ PGA_m_ (0.37), ProfC_m_ (0.42), MMI_m_ (0.45), Spur (0.48), PlanC_m_ (0.52), TWI_m_ (0.55) |
| E | Slope_m_ (0.04), Ridge (0.08), PGA_m_ (0.09), Hollow (0.14), Slope_σ_ (0.17), Spur (0.27), TWI_σ_ (0.33), ProfC_m_ (0.351), PGV_m_ (0.357), Slope (landform class) (0.39), PlanC_σ_ (0.422), MMI_m_ (0.423), PlanC_m_ (0.49)_,_ VRM_m_ (0.52), TWI_m_ (0.59) |

**Supplementary Table S2.** Results of hot spot analysis, described in Step 5 (*cf.* Section 3). For each map, corresponding to the difference of pair of inventories, we list the percentage of slope units in each hot spot class. The percentages in the “Result” column represent the confidence level.

| **Result** | **Inventories** | | |
| --- | --- | --- | --- |
|  | **A-B** | **B-C** | **C-A** |
| Hot Spot 99% | 23.8 | 16.3 | 4.3 |
| Hot Spot 95% | 19.8 | 2.8 | 2.1 |
| Hot Spot 90% | 7.8 | 1.7 | 1.3 |
| Not Significant | 27.6 | 36.4 | 92.3 |
| Cold Spot 90% | 1.1 | 7.8 | 0.0 |
| Cold Spot 95% | 2.1 | 16.8 | 0.0 |
| Cold Spot 99% | 17.8 | 18.3 | 0.0 |

**Supplementary Table S3.** Results of cluster and outlier analysis, for subtracted maps, described in Step 5 (*cf.* Section 3). The number represents the percentage.

| **Result** | **Inventories** | | |
| --- | --- | --- | --- |
|  | **A-B** | **B-C** | **C-A** |
| Not significant | 27.0 | 35.5 | 25.4 |
| Cluster: High | 17.2 | 10.5 | 56.5 |
| High Outlier | 0.3 | 2.8 | 1.6 |
| Low Outlier | 1.8 | 0.8 | 0.2 |
| Cluster: Low | 53.6 | 15.3 | 16.3 |
